# The seroprevalence of SARS‐CoV‐2 infection in Malaysia: 7 August to 11 October 2020

**DOI:** 10.1111/irv.13193

**Published:** 2023-10-01

**Authors:** Zhuo‐Lin Chong, Wan Shakira Rodzlan Hasani, Filza Noor Asari, Eida Nurhadzira Muhammad, Mohd Hatta Abdul Mutalip, Tania Gayle Robert Lourdes, Halizah Mat Rifin, Sarbhan Singh, Ravindran Thayan

**Affiliations:** ^1^ Institute for Public Health, National Institutes of Health, Ministry of Health Malaysia Setia Alam Selangor Malaysia; ^2^ Department of Community Medicine, School of Medical Sciences Universiti Sains Malaysia Kubang Kerian Kelantan Malaysia; ^3^ Institute for Medical Research, National Institutes of Health Ministry of Health Malaysia Setia Alam Selangor Malaysia

**Keywords:** COVID‐19, Malaysia, SARS‐CoV‐2, seroprevalence, WHO‐UNITY studies

## Abstract

**Background:**

From the beginning of the COVID‐19 pandemic until mid‐October 2020, Malaysia recorded ~15,000 confirmed cases. But there could be undiagnosed cases due mainly to asymptomatic infections. Seroprevalence studies can better quantify underlying infection from SARS‐CoV‐2 by identifying humoral antibodies against the virus. This study was the first to determine the prevalence of SARS‐CoV‐2 infection in  Malaysia's general population, as well as the proportion of asymptomatic and undiagnosed infections.

**Methods:**

This cross‐sectional seroprevalence study with a two‐stage stratified random cluster sampling design included 5,131 representative community dwellers in Malaysia aged ≥1 year. Data collection lasted from 7 August to 11 October 2020 involving venous blood sampling and interviews for history of COVID‐19 symptoms and diagnosis. Previous SARS‐CoV‐2 infection was defined as screened positive using the Wantai SARS‐CoV‐2 Total Antibody enzyme‐linked immunosorbent assay and confirmed positive using the GenScript SARS‐CoV‐2 surrogate Virus Neutralization Test. We performed a complex sampling design analysis, calculating sample weights considering probabilities of selection, non‐response rate and post‐stratification weight.

**Results:**

The overall weighted prevalence of SARS‐CoV‐2 infection was 0.49% (95%CI 0.28–0.85) (N = 150,857). Among the estimated population with past infection, around 84.1% (95%CI 58.84–95.12) (N = 126 826) were asymptomatic, and 90.1% (95%CI 67.06–97.58) (N = 135 866) were undiagnosed.

**Conclusions:**

Our study revealed a low pre‐variant and pre‐vaccination seroprevalence of SARS‐CoV‐2 infection in Malaysia up to mid‐October 2020, with a considerable proportion of asymptomatic and undiagnosed cases. This led to subsequent adoption of SARS‐CoV‐2 antigen rapid test kits to increase case detection rate and to reduce time to results and infection control measures.

## 1 INTRODUCTION

Coronavirus disease 2019 (COVID‐19) has rapidly spread globally in an unprecedented manner since it was first detected in December 2019 in China.^1^ On 11 March 2020, the World Health Organization (WHO) declared it a pandemic. By then, 118,319 confirmed cases and 4,292 deaths had been attributed to COVID‐19 globally.^2^ In Malaysia, the first imported cases were detected in January 2020,^3^ and the first local transmission occurred in February 2020.^1^ Together with a few imported cases, these constituted the first wave of the COVID‐19 pandemic in this Southeast Asian nation with more than 30 million population.^4^, ^5^ The second wave of infection started in March 2020, prompting the government to implement strict public health measures such as mandatory face masking and physical distancing, and even a nationwide movement control order (MCO) that restricted international and most local travels, which successfully flattened the curve in early June 2020. Throughout the ensued recovery MCO when all local travels were permitted again, the number of daily reported COVID‐19 cases remained relatively low.^4^ Up until the third wave began in mid‐October 2020, the all‐time cumulative cases were only around 15,000.^4^, ^6^

This count included only cases with laboratory confirmation of infection by reverse transcriptase polymerase chain reaction (RT‐PCR), which in turn was performed largely on suspicious cases with respiratory infection and relevant travel history or close contact with a confirmed case.^1^ This is understandable at the initial stage of the COVID‐19 pandemic, when the disease was not yet known to present asymptomatic or with diverse symptoms.^7^, ^8^ Coupled these with the lack of diagnostic resources and barriers to access healthcare, the incidence figure could be underestimated due to undiagnosed cases, whether symptomatic or asymptomatic.^7^, ^8^, ^9^, ^10^

SARS‐CoV‐2 infection leaves a serological trace in the infected person's body. The presence of binding antibodies towards the receptor‐binding domain (RBD) of the virus indicates past infection, whereas their ability to neutralise the virus indicates that the immunity formed is likely protective.^11^ A survey that examines the seroprevalence of SARS‐CoV‐2 early in the pandemic before the advent of vaccination, when only a relatively small proportion of the population had begun to encounter the wild‐type virus for the first time, can provide a better understanding to its transmission. This proxy of past infection serves as an additional indicator of disease burden that could quantify the extent of COVID‐19 underreporting, inform policymakers of the effectiveness of existing public health measures and lead to the improvement of these measures for better disease prevention and control.^9^, ^10^

During the first year of the pandemic, up to mid‐August 2020, the reported seroprevalence of SARS‐CoV‐2 infection varied widely globally in the range of 0.4–22.1% due to epidemiological and study methodology variations, including the type of serology tests used, which differed in their accuracy.^10^ Around the same time, the meta‐analysed pooled SARS‐CoV‐2 seroprevalence among the general population in the Western Pacific Region was 1.7% (95%CI 0.0–5.0).^12^ In Malaysia, two studies conducted in health facilities located in the country's most urbanised region around mid‐2020 reported zero SARS‐CoV‐2 seroprevalence among healthcare workers and 0.4% among patients with non‐respiratory infections.^13^, ^14^

While these studies pointed to a low COVID‐19 burden in the urban areas of Malaysia during the first few months of the COVID‐19 pandemic, the situation among the general population across the whole nation remained unquantified. A nationally representative community‐based SARS‐CoV‐2 seroprevalence study was necessary to better inform COVID‐19 prevention and control in Malaysia. Furthermore, as the number of infected people continued to increase, a repeated seroprevalence study might be required, preferably with a more efficient diagnostic kit. This study aimed to determine the prevalence of SARS‐CoV‐2 infection and the proportion of asymptomatic and undiagnosed SARS‐CoV‐2 infections in Malaysia and to determine the diagnostic accuracy of a SARS‐CoV‐2 antibody rapid diagnostic test (RTK) for its potential application in repeated seroprevalence studies.

## 2 METHODS

### 2.1 Study design

This study was part of the 2020 National Health and Morbidity Survey (NHMS). The NHMS is an annual cross‐sectional survey conducted by the Institute for Public Health (IPH), Ministry of Health, Malaysia. It surveys the nationwide general population on a different health‐related topic each year using structured questionnaires and usually minimally invasive clinical measurement (capillary blood sampling) to inform health policymaking. In 2020, the subject of interest was infectious diseases.

The methods of NHMS 2020 have been described in detail elsewhere.^5^ In brief, this survey was designed to represent non‐institutionalised population in Malaysia, irrespective of citizenship. We used a two‐stage stratified random cluster sampling design, with the primary stratum being states and territories in Malaysia (target population size range = 181,859–6,239,037), while the secondary stratum consisted of urban and rural areas within the primary stratum. We acquired from the Department of Statistics, Malaysia (DOSM), the sampling frame that arbitrarily divided the country into >90,000 areas called enumeration blocks (EB), each containing 80–120 living quarters (LQ) or 500–600 non‐institutionalised community‐dwelling individuals. EBs served as the primary sampling unit (PSU), and LQs were the secondary sampling unit (SSU). In total, we selected 113 EBs nationwide, with 83 in Peninsular Malaysia, 13 in Sabah and 17 in Sarawak. From each selected EB, 20 LQs were randomly chosen.

For SARS‐CoV‐2 seroprevalence specifically, we included all permanent residents aged 1 year and above in the selected LQ. We excluded LQs that housed temporary occupants and individuals with contraindications to venipuncture. We expected an overall sample size of at least 5,000 participants to achieve an estimated SARS‐CoV‐2 seroprevalence in Malaysia of 1.0% with a precision of ±0.3% at 95% confidence.^5^, ^15^

### 2.2 Field data collection

To ensure standardisation and facilitate comparisons, we adapted the WHO UNITY COVID‐19 seroepidemiological study protocol by including all questions required to capture necessary variables for data sharing with the WHO.^9^ Between 7 August and 11 October 2020 (the window period between the second and third waves of the COVID‐19 pandemic in Malaysia), trained data collectors visited eligible participants and interviewed consented (and assented for <18 years) ones face‐to‐face using mobile tablets with a structured questionnaire that captured exposure variables such as basic sociodemographic background, COVID‐19 epidemiology including previous COVID‐19 diagnosis, and history of COVID‐19‐like symptoms in the year 2020. Subsequently, trained phlebotomists collected up to 5 ml of venous blood from each participant in a gel tube with clot activator.

Using residual blood specimens from the syringe, phlebotomists performed the Wondfo One Step COVID‐19 (SARS‐CoV‐2 Antibody) Test (Guangzhou Wondfo Biotech, China), an immunochromatographic rapid test kit (RTK) according to the manufacturer's instructions, as described previously.^16^ Within 15–20 min of the test, two independent interpreters read the results if the control line became visible, indicating that the test was valid. Each independently reported any visible test line as a positive result, indicating the presence of anti‐SARS‐CoV‐2 immunoglobulin M or G (IgM/IgG), that is, a previous infection, and vice versa. A third person participated in the interpretation process to resolve any discrepancies.

After the venous blood specimens clotted, trained laboratory technicians spun them onsite using a portable centrifuge and chilled them immediately in a portable freezer at 2–8°C. This cold chain was maintained and monitored with a digital temperature logger all through the transportation process until the specimens reached a virology laboratory in the Institute for Medical Research, Malaysia, where trained laboratory personnel aliquoted and stored them at −80°C until adequate samples were accumulated for subsequent serology tests.

### 2.3 Laboratory tests and case definition of previous SARS‐CoV‐2 infection

1. Wantai SARS‐CoV‐2 Total Antibody Enzyme‐Linked Immunosorbent Assay (ELISA)

Trained laboratory technicians tested serum specimens collected from all participants using the Wantai SARS‐CoV‐2 Total Antibody ELISA kit (Beijing Wantai Biological Pharmacy Enterprise Co., Ltd., China) (sensitivity: 89–99%, specificity: 99–100%), according to the manufacturer's instructions, as described elsewhere.^17^, ^18^ For any test to be valid, the absorbance values of all controls must be within acceptable limits, as per quality control. We then calculated the cut‐off value (COV) by adding 0.16 to the average absorbance of three negative controls. In our study, we interpreted the ratio of the specimen's absorbance to COV as positive if ≥1.0, indicating the presence of anti‐SARS‐CoV‐2 binding IgM/IgG; negative if <0.8; and borderline if ≥0.8 to <1.0.^17^ We repeated all specimens with borderline results.

2 SARS‐CoV‐2 surrogate Virus Neutralization Test (sVNT) Kit

We tested all specimens with borderline and positive ELISA results on cPass™ SARS‐CoV‐2 Neutralization Antibody Detection Kit (Genscript Biotech, United States) (sensitivity: 99%, specificity: 100%) in the laboratory, following the steps described elsewhere.^19^ According to quality control, all controls must have an average optical density (OD) within acceptable limits. We calculated the percentage of neutralization/inhibition (inhibition%) for each specimen using a formula: (1 − OD of a specimen/average OD of all negative controls) × 100%.^19^ In our study, we interpreted the inhibition% as positive if ≥20%, indicating the presence of anti‐SARS‐CoV‐2 neutralising antibodies, and negative if <20%.^19^

### 2.4 Variables definition

The main dependent variable was the status of previous SARS‐CoV‐2 infection according to laboratory tests. We defined a participant as a case of previous SARS‐CoV‐2 infection if the participant tested positive on ELISA first and then confirmed positive using the sVNT kit.^14^ The second dependent variable was the status of previous SARS‐CoV‐2 infection according to onsite RTK.

Independent variables were age, sex, location (urban or rural), history of COVID‐19 diagnosis and history of COVID‐19‐like symptoms. Symptom status was defined as at least one self‐reported COVID‐19‐like symptom since 1 January 2020 (fever, sore throat, runny nose, cough, shortness of breath, chills, vomiting, nausea, diarrhoea, headache, muscle ache, loss of smell, loss of taste and fatigue). The diagnosis status was self‐reported using the screening question, ‘Have you ever been diagnosed as a COVID‐19 patient?’, which in turn followed the early definition of confirmed cases, that is, a positive RT‐PCR.

### 2.5 Data analysis

We used a complex sampling design (weighted analysis) to ensure the representativeness of Malaysia's general population in terms of age group, sex and location. We computed the seroprevalence point estimate and 95% confidence interval (95%CI), as confirmed by the laboratory tests in the general population and its subgroups, and compared different subgroups using the Rao‐Scott adjusted chi‐square test. Next, we stratified the positive cases according to their diagnosis and symptom status to compute the proportion point estimate and 95%CI for each status. Lastly, using standard formulas, we computed the diagnostic accuracy of the RTK (the index test); that is, sensitivity, specificity, positive predictive value (PPV) and negative predictive value (NPV), as compared to the status of previous SARS‐CoV‐2 infection according to laboratory tests (the reference standard). All data analyses were performed using IBM SPSS Statistics for Windows version 25 (IBM, USA) (detailed data analysis in the supporting information).^20^

## 3 RESULTS

A total of 6,818 individuals were eligible for this study. Among them, 5,131 eventually provided viable specimens for laboratory tests, with a response rate of 75.3%. Most participants aged ≥18 years (74.4%), were female (52.3%) and lived in an urban setting (53.9%). After applying complex sampling design analysis, the total estimated population in Malaysia represented by the 5,131 participants was 30,763,427 people (Table 1).

**TABLE 1:** Sociodemographic characteristics of 5,131 study participants recruited from 7 August to 11 October 2020 and the estimated population in Malaysia they represented

Socio‐demographic characteristics		Unweighted count of study participants, n (%)	Estimated population in Malaysia^a^, N (%)
Overall		5,131 (100.0)	30,763,427 (100.0)
Age group	1–17 years	1,312 (25.6)	8,063,772 (26.2)
	≥18 years	3,819 (74.4)	22,699,654 (73.8)
Sex	Male	2,450 (47.7)	15,948,188 (51.8)
	Female	2,681 (52.3)	14,815,239 (48.2)
Location	Urban	2,765 (53.9)	23,657,065 (76.8)
	Rural	2,366 (46.1)	7,106,362 (23.2)

^a^ Complex sampling design analysis was applied to calculate estimated population (adjusted for design weight, non‐response rate and post‐stratification weight, which was adjusted based on the Malaysian population projections for 2020). The estimated population has a degree of uncertainty. Detailed estimation of the population is given in their 95%CI (Table S1 in the supporting information).

The weighted seroprevalence of SARS‐CoV‐2 infection in Malaysia between 7 August and 11 October 2020 was 0.49% (95%CI 0.28–0.85) (n = 25, N = 150,857). The prevalence was significantly lower (*p* = 0.003) among those aged <18 years at 0.10% (95%CI 0.03–0.35) (n = 3, N = 8,264), as compared to 0.63% (95%CI 0.36–1.11) (n = 22, N = 142,593) among those aged ≥18 years. There was no significant difference in the prevalence between males, 0.55% (95%CI 0.27–1.11) (n = 13, N = 88,484), and females, 0.42% (95%CI 0.16–1.10) (n = 12, N = 62,373), as well as between urban, 0.55% (95%CI 0.29–1.02) (n = 16, N = 129,958), and rural dwellers, 0.29% (95%CI 0.14–0.60) (n = 9, N = 20,899) (Table 2).

**TABLE 2:** The weighted prevalence of previous SARS‐CoV‐2 infection in Malaysia by sociodemographic characteristics (from 7 August to 11 October 2020)

Socio‐demographic characteristics	Previous SARS‐CoV‐2 infection^a^		Weighted prevalence of SARS‐CoV‐2 infection^b^, % (95%CI)	*p* value^c^
	Unweighted count, n	Estimated population^b^, N		
Overall	25	150,857	0.49 (0.28–0.85)	‐
Age group				
1–17 years	3	8,264	0.10 (0.03–0.35)	0.003
≥ 18 years	22	142 593	0.63 (0.36–1.11)	
Sex				
Male	13	88,484	0.55 (0.27–1.11)	0.660
Female	12	62,373	0.42 (0.16–1.10)	
Location				
Urban	16	129,958	0.55 (0.29–1.02)	0.187
Rural	9	20,899	0.29 (0.14–0.60)	

^a^ Previous SARS‐CoV‐2 infection was defined as a positive test for both Wantai SARS‐CoV‐2 Total Antibody ELISA and cPass SARS‐CoV‐2 sVNT.

^b^ Complex sampling design analysis was applied to calculate estimated population and weighted prevalence (adjusted for design weight, non‐response rate and post‐stratification weight, which was adjusted based on the Malaysian population projections for 2020). The estimated population has a degree of uncertainty. Detailed estimation of the population is given in their 95%CI (Table S1).

^c^ *P* value was computed using Rao–Scott adjusted chi‐square statistics.

The weighted proportion of symptomatic SARS‐CoV‐2 infection was 15.9% (95%CI 4.9–41.2) (n = 8, N = 24,031), much lower than 84.1% (95%CI 58.8–95.1) (n = 17, N = 126,826) of asymptomatic infection. On the other hand, only 9.9% (95%CI 2.4–32.9) (n = 5, N = 14,992) of SARS‐CoV‐2 infections were estimated to be previously diagnosed, while an estimate of 90.1% (95%CI 67.1–97.6) (n = 20, N = 135,866) went undiagnosed (Table 3).

**TABLE 3:** The weighted proportion of previous SARS‐CoV‐2 infection in Malaysia by symptom and diagnosis status (from 7 August to 11 October 2020)

Characteristics	Previous SARS‐CoV‐2 infection		Weighted proportion of SARS‐CoV‐2 infection^a^, % (95%CI)
	Unweighted count, n	Estimated population^a^, N	
Symptomatic	8	24 031	15.9 (4.9–41.2)
Asymptomatic	17	126 826	84.1 (58.8–95.1)
Diagnosed	5	14 992	9.9 (2.4–32.9)
Undiagnosed	20	135 866	90.1 (67.1–97.6)

^a^ Complex sampling design analysis was applied to calculate estimated population and weighted proportion (adjusted for design weight, non‐response rate, and post‐stratification weight, which was adjusted based on the Malaysian population projections for 2020). The estimated population has a degree of uncertainty. Detailed estimation of the population is given in their 95%CI (Table S1).

Of the 5,131 individuals tested using laboratory tests, Wondfo RTK results were available for 5,118. There were four positive RTK results, two of which were true positives. Of the 5,114 negative RTK results, the number of true negatives was 5,091. The RTK had a sensitivity and specificity of 8.0% (95%CI 1.4–22.7) and 99.9% (95%CI 99.9–100.0), and its positive and negative predictive values were 50.0% (95%CI 10.7–89.3) and 99.6% (95%CI 99.3–99.7), respectively (Table 4).

**TABLE 4:** The 2 × 2 table for the computation of the diagnostic accuracy of Wondfo One Step COVID‐19 (SARS‐CoV‐2 Antibody) rapid test kit

Wondfo SARS‐CoV‐2 antibody RTK	Previous SARS‐CoV‐2 infection^a^		Total	Positive predictive value (PPV)	Negative predictive value (NPV)
	Present	Absent			
Positive	**2**	2	**4**	50.0 (10.7, 89.3)	‐
Predictive value	23	**5,091**	**5,114**	‐	99.6 (99.3, 99.7)
PPV	**25**	**5,093**	5118		
**Sensitivity**	8.0 (1.4, 22.7)	‐	‐	**Diagnostic accuracy, % (95%CI)** ^b^	
**Specificity**	‐	99.9 (99.9, 100.0)			

^a^ Previous SARS‐CoV‐2 infection was defined as a positive test for both Wantai SARS‐CoV‐2 Total Antibody ELISA and cPass SARS‐CoV‐2 sVNT.

^b^ Each diagnostic accuracy estimate was calculated by dividing the number of positive or negative RTK results by the total number of tests performed (both figures bolded) in the respective columns or rows.

## 4 DISCUSSION

We report a low overall pre‐variant and pre‐vaccination SARS‐CoV‐2 seroprevalence (0.49%) between 7 August and 11 October 2020 in Malaysia. This seroprevalence was six times higher (*p* = 0.003) in the population aged 18 years and above. No difference was found between the sexes and the level of urbanisation. A high proportion (84.1–90.1%) of previously infected individuals was asymptomatic and undiagnosed. On the other hand, the SARS‐CoV‐2 antibody RTK had a low sensitivity of <10% and close to perfect specificity and NPV, whereas its PPV estimate was moderate but lacked precision.

In the first year of the COVID‐19 pandemic, before the emergence of variants and mass vaccination, the seroprevalence of SARS‐CoV‐2 infection varied widely between countries due mainly to differences in the timing of the study relative to the spread of COVID‐19 among countries.^10^ As the spread of COVID‐19 can differ even within countries, provinces or communities, bias in participant selection would also overestimate or underestimate the overall seroprevalence.^10^ On the other hand, serological tests used to detect anti‐SARS‐CoV‐2 antibodies also differ in their diagnostic accuracy, resulting in measurement bias. Less sensitive diagnostic tests, such as lateral flow RTK, detect fewer positives and yield lower seroprevalence as compared to more sensitive laboratory‐based tests, such as ELISA^10^, ^21^, ^22^ However, more sensitive tests, in combination with a more stringent case definition of a previous infection, would also yield a lower seroprevalence.^5^ Hence, similar national level studies using probability sampling and laboratory‐based diagnostics would be more comparable to our study. Only two countries neighbouring Malaysia reported such studies, namely, Thailand with a SARS‐CoV‐2 seroprevalence of 0.4% (95%CI 0.1–1.0%) and Indonesia ‐ 20.5% (95%CI 19.7–21.3%), both conducted later in mid‐December 2020.^23^ At that point of time, two months had lapsed since the end of this study that coincided with the beginning of the third wave on the COVID‐19 epidemic curve in Malaysia.^4^ The SARS‐CoV‐2 seroprevalence in Malaysia in December 2020 would have been higher than our 0.49% point estimate and likely situated between the estimates of these two neighbouring countries.

The proportions of asymptomatic and undiagnosed SARS‐CoV‐2 infections also differed among studies. Asymptomatic infections ranged widely from 3.7% to 87.5% among confirmed COVID‐19 cases, according to a systematic review up to 4 February 2021.^24^ The main reasons for the heterogeneity could be variations in study design, such as the timing of symptom ascertainment and the study population, where repeated symptom ascertainment at a later time as opposed to only at the initial test, and studies among contacts only instead of the general population tended to report a lower proportion of asymptomatic infection.^25^ Our study was conducted among the general population and, hence, a higher weighted proportion of asymptomatic infections. In addition, recall bias could have contributed to fewer reports of symptoms when assessed retrospectively in this study. Future studies using a prospective design with repeated symptom assessments may provide better estimates.

In terms of the proportion of undiagnosed COVID‐19 cases, apart from the study population and the accuracy of serological tests used, the main reasons for heterogeneity across studies could be the fundamental differences between countries and administrative entities in their definition of COVID‐19 cases and contacts, the aggressiveness of contact tracing, testing availability and strategy, and other factors that would affect the yield of case detection relative to actual infection.^26^, ^27^, ^28^, ^29^, ^30^ On the day we concluded our blood collection, 11 October 2020, Malaysia recorded 15,657 confirmed COVID‐19 cases.^6^ This cumulative number of confirmed cases approximated our 14,992 point estimate of previously diagnosed COVID‐19 cases, providing confidence to this estimate and also to the estimate and proportion of the undiagnosed cases. This led to subsequent adoption of a positive SARS‐CoV‐2 antigen RTK result to the COVID‐19 case definition in Malaysia to increase case detection rate and reduce time to results and infection control measures.^31^

Our evaluation of the SARS‐CoV‐2 total antibody RTK found it to have 99.9% specificity and 8.0% sensitivity. Although this RTK did not produce false positives, it produced 92 false negatives and only detect 8 out of 100 previous infections. Comparatively, the sensitivity and specificity of other SARS‐CoV‐2 total antibody RTKs ranged around 18.4–100.0% and 80.6–100.0%, respectively.^22^ Although a direct comparison of diagnostic accuracy across different studies cannot be completed without assessing the underlying biases stemming from heterogeneity in different studies,^32^ the low sensitivity of this particular RTK precludes its application in future seroprevalence studies.^22^ Any COVID‐19 seroprevalence study using RTK should be based on a proper prior evaluation and adjusted for measurement bias to obtain a more accurate seroprevalence estimate.^27^, ^33^

This study was conducted in 2020 between the second and third waves of the COVID‐19 pandemic in Malaysia.^4^ The SARS‐CoV‐2 seroprevalence was low but significantly higher among adults, likely due to higher mobility. As a result of increased mobility and the emergence of more transmissible SARS‐CoV‐2 variants,^4^ subsequent epidemic waves became many times larger in magnitude. On the other hand, Malaysia initiated its mass COVID‐19 vaccination programme at the end of February 2021.^34^ These developments would lead to a substantial increase in SARS‐CoV‐2 seroprevalence since the end of our study, albeit antibody decay might slightly negate that. Repeated seroprevalence studies were initially deemed necessary by the policy makers to monitor the progress of pandemics and vaccination programmes. However, in view of the risk of COVID‐19 transmission and the significantly higher price tag this representative community‐based survey carried, and also the lack of a more efficient yet accurate antibody RTK to reduce the cost, the IPH instead proposed and subsequently conducted another SARS‐CoV‐2 seroprevalence study in 2021 with residual blood donor specimen using the same ELISA. To better inform the vaccination programme with local data, Malaysia also commissioned several seroconversion studies in 2021, with IPH following the largest cohort of general population who received different COVID‐19 vaccines over a year.

The main strength of this study was its complex sampling design, which ensured the representativeness of Malaysia's general population. As a result, the number of people with previous SARS‐CoV‐2 infection and its breakdown into different subgroups could be estimated. Second, confirmation of the diagnosis with a neutralisation test makes our seroprevalence estimate more accurate in reflecting the level of protection against SARS‐CoV‐2 in our population. However, the opposite of this more specific case definition is that our results are a more conservative estimate of previous exposure to the virus. We recognise that the self‐reported nature of the symptom status is subject to recall bias, which may be a limitation of our study. As for other data that could subject to the same bias, we verified them with documented proof where possible.

## 5 CONCLUSIONS

The prevalence of SARS‐CoV‐2 in Malaysia was low (0.49%) in the first year of the COVID‐19 pandemic before the emergence of variants and mass vaccination but higher (0.63%) among adults. As a large proportion (84.1–90.1%) of SARS‐CoV‐2 infection was asymptomatic and undiagnosed, an increase in SARS‐CoV‐2 screening and testing was necessary and reflected in Malaysia's testing strategy. The COVID‐19 pandemic continues to evolve amid mass vaccination and booster programmes to control it. This progress can be monitored through repeated SARS‐CoV‐2 serosurveillance with an improved efficiency in the future.

## AUTHOR CONTRIBUTIONS

**Zhuo Lin Chong:** Conceptualization; data curation; formal analysis; funding acquisition; methodology; visualization; writing—original draft. **Wan Shakira Rodzlan Hasani:** Data curation; formal analysis; methodology; visualization; writing—review and editing. **Filza Noor Asari:** Validation; writing—original draft. **Eida Nurhadzira Muhammad:** Project administration; validation. **Mohd Hatta Abdul Mutalip:** Conceptualization; funding acquisition; methodology; project administration; writing—review and editing. **Tania Gayle Robert Lourdes:** Investigation; visualization. **Halizah Mat Rifin:** Investigation; visualization. **Sarbhan Singh:** Validation; writing—review and editing. **Ravindran Thayan:** Methodology; validation.

## CONFLICT OF INTEREST STATEMENT

All authors declared no conflict of interest.

### PEER REVIEW

The peer review history for this article is available at https://www.webofscience.com/api/gateway/wos/peer-review/10.1111/irv.13193.

## ETHICS STATEMENT

Ethics approval was obtained from the Medical Research & Ethics Committee, Ministry of Health Malaysia: NMRR‐20‐1166‐55133 (IIR). Written informed consent was obtained from all participants. Additional written assent was obtained for participants <18 years old, where possible. No material was reproduced from other sources.

## Supporting information


**Table S1.** Detailed data analysis and estimation.

## Data Availability

Data are available on request due to privacy/ethical restrictions.
