# Supplementary material for: The seroprevalence of SARS‐CoV‐2 infection in Malaysia: 7 August to 11 October 2020
Source: Influenza Other Respir Viruses. 2023 Oct 1;17(10):e13193. doi: 10.1111/irv.13193 (PMC10542612; doi:10.1111/irv.13193)
Supplement: Supplementary file 1 — Table S1. Detailed data analysis and estimation. [file IRV-17-e13193-s001.docx]

**Detailed Data Analysis**

To ensure the representativeness of Malaysia's general population in terms of age, sex, and location, we employed a complex sampling design (weighted analysis). We calculated sample weights for each individual, taking into account varying probabilities of selection (design weight), non-response rate, and post-stratification weight, adjusted according to Malaysian population projections based on Department of Statistics, Malaysia (DOSM) 2020.^1^

The weight estimation formula used was W_final_ = W_1_ × W_2_ × F × PS, where W_1_ represented the inverse probability of selecting the enumeration blocks (EB), W_2_- the inverse probability of selecting the living quarters (LQ) within selected EBs, F- the non-response adjustment factor for individuals and LQs, and PS- the post-stratification adjustment factor calculated by gender, strata, age, and ethnicity.

The detailed information on response rates for individuals and LQs in each state has been described elsewhere.^2^ Using these weighted analyses, we computed the seroprevalence point estimate and 95% confidence interval (95% CI) for the overall study population and each of its subgroups.

To account for the complex survey design, we used the Rao-Scott adjusted chi-square statistic to examine the associations between sociodemographic characteristics and the prevalence of SARS-CoV-2 infection. This statistical approach allowed for accurate inference and valid hypothesis testing, taking into consideration the complex sampling structure of the study.^3^ Two-sided P values of <0.05 were considered statistically significant.

Next, we stratified the positive cases based on their symptom and diagnosis status to compute the proportion point estimate and 95% confidence interval (95% CI) for each status.

To evaluate the diagnostic accuracy of the index test, the Wondfo One Step COVID-19 (SARS-CoV-2 Antibody) rapid test kit (RTK), we compared its test results with the reference standard, i.e, the dependent variable of previous SARS-CoV-2 infection, which is defined as positive if tested positive on Wantai SARS-CoV-2 Total Antibody ELISA kit (Beijing Wantai Biological Pharmacy Enterprise Co., Ltd., China) first, and then confirmed positive using the cPass™ SARS-CoV-2 Neutralization Antibody Detection Kit (Genscript Biotech, USA). We calculated sensitivity (true positive rate), specificity (true negative rate), positive predictive value (PPV), and negative predictive value (NPV) using the following formulas:^4^

• Sensitivity = True Positives / (True Positives + False Negatives)

• Specificity = True Negatives / (True Negatives + False Positives)

• PPV = True Positives / (True Positives + False Positives)

• NPV = True Negatives / (True Negatives + False Negatives)

All data analyses were performed using IBM Statistical Package for the Social Sciences (SPSS) software for Windows, Version 25.0.^5^

**Detailed Estimation**

Complex sampling design analysis allowed for the computation of the weighted prevalence and proportion from the estimated population and subpopulation with previous SARS-CoV-2 infection as defined by the laboratory results in this study, by dividing them over the total estimated population or respective subpopulation accordingly. But all estimated population figures are still an estimation in itself and contains a certain degree of uncertainty. This uncertainty is illustrated in the 95% CI of all the estimated population and subpopulation presented in-text(Table S1).

**Table S1** Detailed data analysis and estimation

| **Category** | | **Unweighted count of study participants,**  **n (%)** | **Estimated**  **population**  **in Malaysia,**  **N (%)** |
| --- | --- | --- | --- |
| *Total estimated population and subpopulation*  *(detailed estimation for Table 1 in the main article)* | | | |
| Overall | | 5131 | 30,763,427 (26,604,948 – 34,921,905) |
| Age group | 1-17 years | 1312 | 8,063,772 (6,622,160 – 9,505,385) |
|  | ≥ 18 years | 3819 | 22,699,654 (19,564,871 – 25,834,438) |
| Sex | Male | 2450 | 15,948,188 (13,773,896 – 18,122,480) |
|  | Female | 2681 | 14,815,239 (12,669,553 – 16,960,924) |
| Location | Urban | 2765 | 23,657,065 (19,605,081 – 27,709,049) |
|  | Rural | 2366 | 7,106,362 (6,171,285 – 8,041,439) |
| *Estimated population and subpopulation with previous SARS-CoV-2 infection*  *(detailed estimation for Table 2 in the main article)* | | | |
| Overall | | 25 | 150,857 (64,006 – 237,709) |
| Age group | 1-17 years | 3 | 8,264 (-1,772 – 18,299) |
|  | ≥ 18 years | 22 | 142,593 (55,905 – 229,282) |
| Sex | Male | 13 | 88,484 (26,078 – 150,890) |
|  | Female | 12 | 62,373 (-1,428 – 126,174) |
| Location | Urban | 16 | 129,958 (44,333 – 215,583) |
|  | Rural | 9 | 20,899 (6,354 – 35,444) |
| *Estimated population with previous SARS-CoV-2 infection by symptom and diagnosis status (detailed estimation for Table 3 in the main article)* | | | |
| Symptomatic | | 8 | 24,031 (1,632 – 46,430) |
| Asymptomatic | | 17 | 126,826 (50,673 – 202,978) |
| Diagnosed | | 5 | 14,992 (-3,121 – 33,104) |
| Undiagnosed | | 20 | 135,866 (61,686 – 210,046) |

**References**

1. Department of Statistics Malaysia. Population Quick Info. https://pqi.stats.gov.my/searchBI. php?tahun=2020&kodData=3&kodJadual=1&kodCiri=7&kodNegeri=00 Accessed Jul 27, 2023.

2. Institute for Public Health Malaysia. National Health and Morbidity Survey (NHMS) 2020: Communicable Diseases Volume I. http://iku.gov.my/images/IKU/Document/REPORT/2020/report/1_Report_NHMS2020_Vol-1.pdf

Accessed Dec 20, 2021.

3. Rao JNK, Scott AJ. On Chi-Squared Tests for Multiway Contingency Tables with Cell Proportions

Estimated from Survey Data. Ann Stat. 1984;12(1):46-60.

4. Chong ZL, Sekaran SD, Soe HJ, et al. Diagnostic accuracy and utility of three dengue diagnostic tests for the diagnosis of acute dengue infection in Malaysia. BMC Infect Dis. 2020;20(1):210.

5. IBM Corp. IBM SPSS Statistics for Windows, Version 25.0. Armonk, NY: IBM Corp. Released

2017.
